# Musculo-Invasive Urachal Transitional Cell Carcinoma (TCC): Is It Really Sinister and Different?

**DOI:** 10.7759/cureus.98301

**Published:** 2025-12-02

**Authors:** Maniyur Raghavendran, Maniyur Shreya, Nirmall U, Benaganahalli Sandeep, Vinay N Kaushik

**Affiliations:** 1 Urology, Apollo Hospitals, Mysuru, IND; 2 General Surgery, Krishna Rajendra Hospital (KRH), Mysuru, IND; 3 General Surgery, Apollo Hospitals, Mysuru, IND; 4 Radiology, Apollo Hospitals, Mysuru, IND

**Keywords:** high grade bladder tcc, high grade urachal tcc, margin positivity, musculo-invasive bladder tcc, musculo-invasive urachal tcc, urachal urothelial carcinoma

## Abstract

Urachal remnants are found in a third of adults, commonly at the dome. They can be found anywhere along the bladder midline, including the anterior and posterior walls. As a single entity, urachal carcinomas are rare, comprising 0.01% of all adult cancers and 0.35% of all bladder cancers. The commonest urachal carcinoma is adenocarcinoma, but 5% of urachal growths are urothelial. Whenever a case of urachal carcinoma is discussed, it is presumed to be an adenocarcinoma, but people forget the possibility of a transitional cell carcinoma (TCC) occurring in the urachus. Also, there is confusion about whether it is a primary urachal TCC or has spread from the adjacent bladder mucosa. It is surprising to note that to date, only 13 cases of primary urachal TCC have been described, and hence, not much is known about this rare tumor. As of today, the factors affecting the prognosis in this rare tumor are, namely, four: grade, stage, margin status, and musculo-invasive nature. A thorough search of the literature did not reveal a single case that encompasses all four prognostic factors. To the best of our knowledge, ours is the first case of high-grade musculo-invasive urachal TCC presenting with early-stage disease. This enabled us to dissect which of the four factors, namely grade, stage, margin status, and muscle invasion, influences the prognosis. We would like to discuss the clinical features of the distinction between primary and secondary urachal TCC. We would also like to specially emphasize and discuss whether there is any behavioral change when compared to the more commonly occurring adjacent bladder TCC.

## Introduction

The urachus is a duct connecting the allantois to the early fetal bladder. It is three-layered with a luminal layer lined by cuboidal or transitional epithelium, a submucosal connective tissue layer, and an outer smooth muscle layer [1].

In 95% of younger individuals with urachal remnant tissue, the epithelium is lined by urothelial cells, but in older people, the lining layer of the urachal remnants constitutes of more glandular lining cells [2].

As the bladder descends into the pelvis, the urachus is progressively stretched, and the lumen becomes obliterated. However, in 30% of people, the vestiges of the urachus remain and can be identified in the bladder wall, especially in the dome and rarely in the posterior wall [2].

A total of 10% of urachal remnants communicate with the bladder lumen, suggesting bladder cancer can spread into the remnant lumen by continuity. This constitutes secondary urachal TCC [3].

Urachal remnants can give rise to urachal carcinoma. They constitute less than 1% of all bladder cancers. The most common of these is adenocarcinoma, but 5-10% can be of urothelial origin. This group of tumors is the primary urachal TCC, our index case in this report [4].

To summarize, urachal remnant involvement by urothelial carcinoma can be due to spread in continuity from adjacent bladder TCC due to persistent communication. These are considered secondary urachal TCC. They can also present as a separate tumor focus in the urachus along with a second concomitant bladder TCC. Though these tumors are not in continuity and represent two distinct tumors, they are still considered secondary urachal TCC.

An isolated urachal tumor with no contiguous or noncontiguous tumor in the bladder, as seen in our case, has also been reported, albeit rarely. These are labelled as primary urachal TCC, and to the best of our knowledge, only 13 cases have been described as of date. In this case report, we would like to discuss a case that we concluded was a primary urachal TCC and emphasize why we came to that conclusion.

Several staging systems are used now, with the most used being the Sheldon staging system and Ashley’s Mayo staging system [5,6].

Pederson et al. opined that high stage and positive surgical margins are the strongest prognostic indicators [7]. They could not comment on the prognostic role of musculo-invasive status, as their tumor was superficial and noninvasive. To the best of our knowledge, ours is the first reported case of high-grade musculo-invasive urachal TCC presenting at an earlier, locally confined stage. Hence, we wanted to look into our case and see which among the 4 factors-grade, stage, margin positivity, and musculo-invasiveness-affected the prognosis the most [7].

## Case presentation

**Figure 1:**
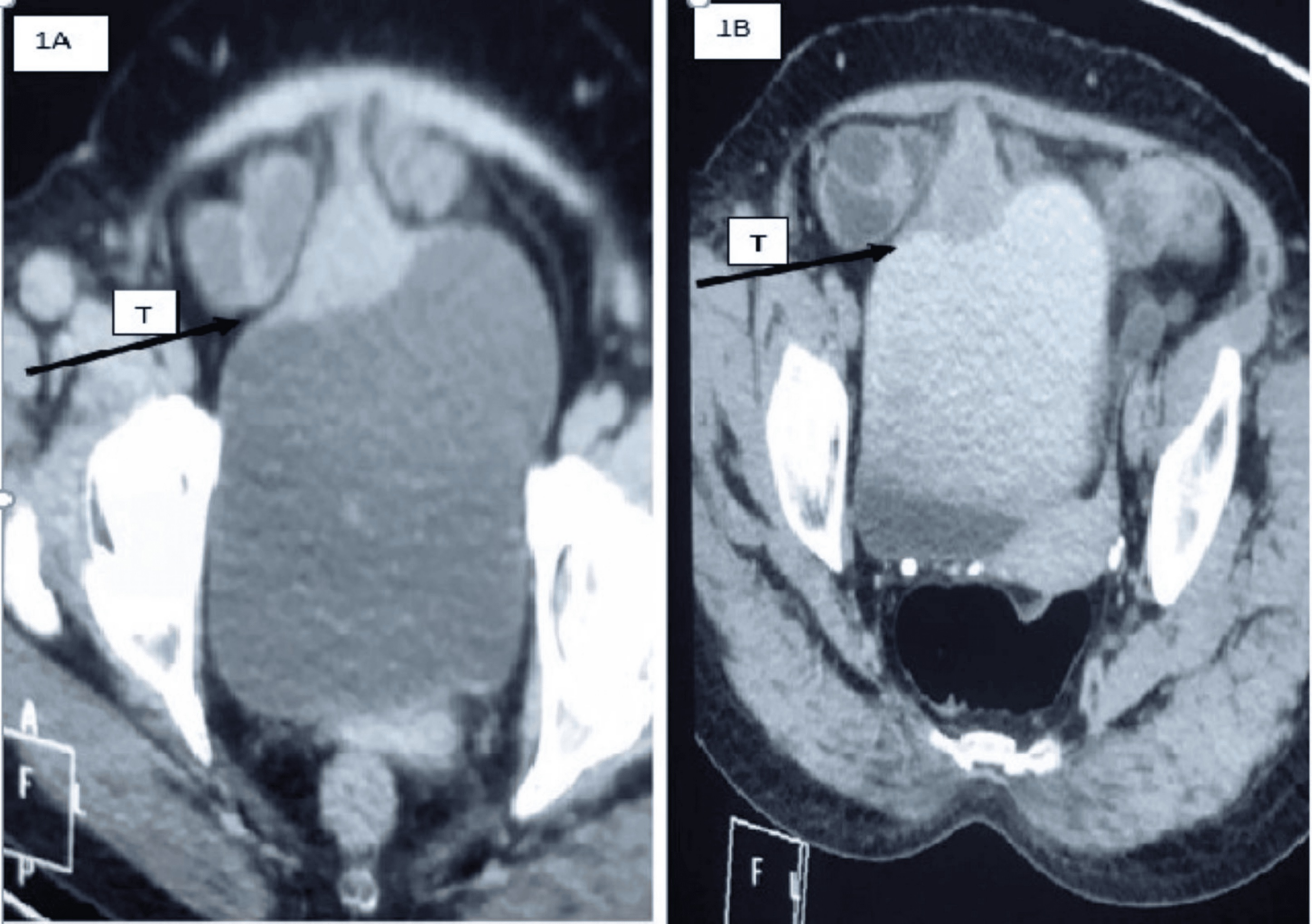
Contrast-enhanced CT images showing urachal growth 1a and 1b depicting plain and CECT images confirming a urachal growth (T) (black arrowhead)

**Figure 2:**
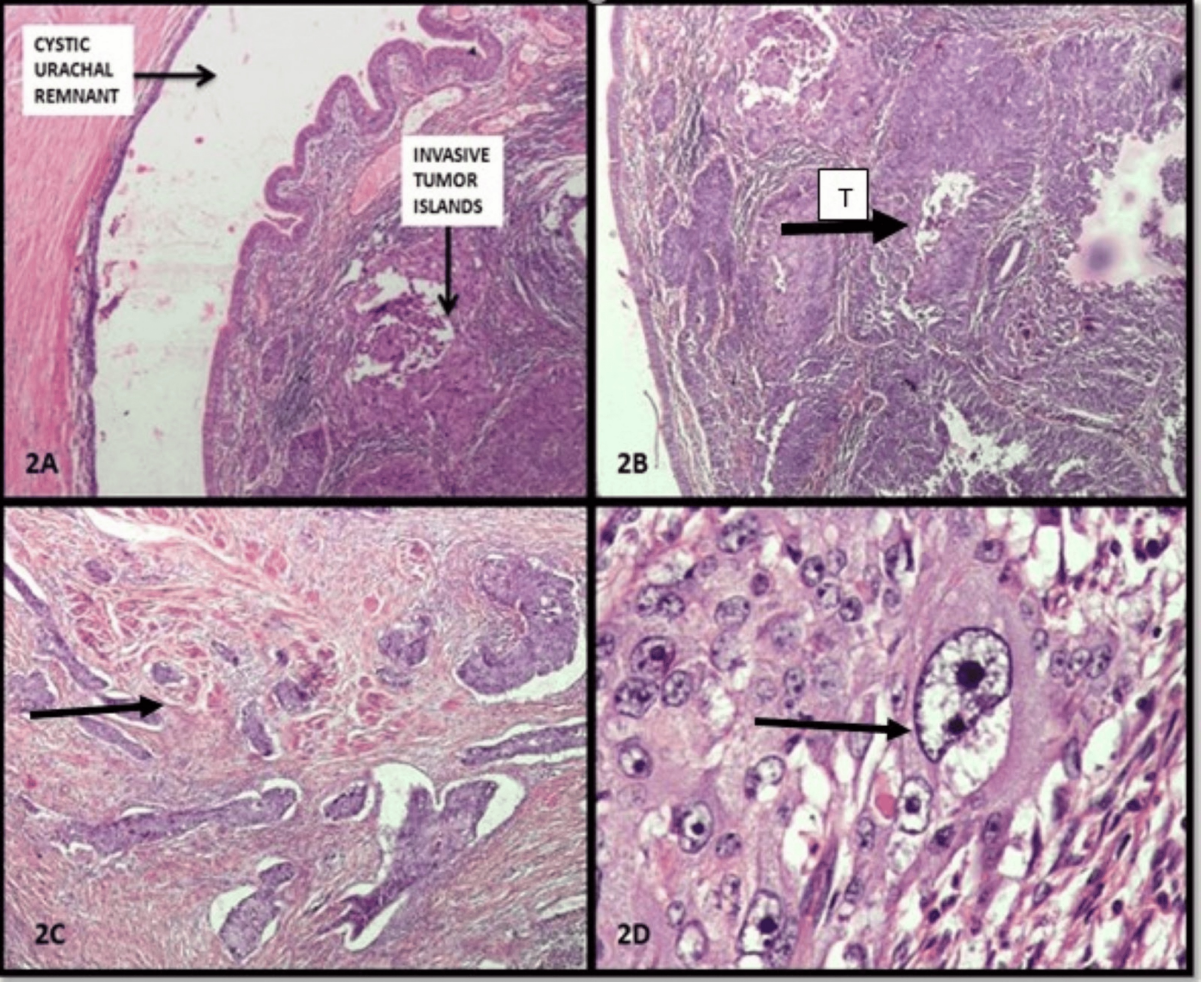
Histopathological pictures of the tumor 2A- Arrowhead showing cystic urachal remnant with overlying invasive tumor (H&E x 40), 2B- Urothelial lining overlying islands and nests of urothelial carcinoma (black arrowhead with letter T) (H&E x 40), 2C- Urothelial carcinoma invading the muscle (black arrowhead) (H&E x 40), 2D- High-grade nuclear features with pleomorphic nuclei, prominent nucleoli, and scant cytoplasm (black arrowhead) (H&E x 400). Note must be made of the absence of intense inflammatory and desmoplastic reactions.

**Figure 3:**
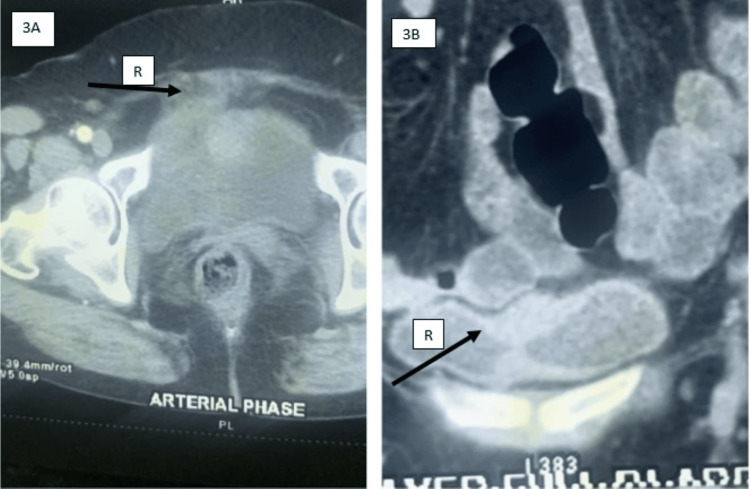
CECT images during the follow-up period Follow-up CECT (3A and 3B) showing recurrence confined to the bladder in sagittal and coronal sections (black arrowhead with letter R)

A 55-year-old female presented with a slowly enlarging mass in the umbilical region. She had no urinary symptoms at presentation. There were no other significant comorbidities. Her past history was unremarkable. She was a nonsmoker and nonalcoholic. Clinical examination revealed a hard 5 cm palpable mass. Contrast-enhanced CT (CECT) (Figures 1a, 1b) confirmed urachal origin with no distant metastasis. The entire bowel was normal. Cystoscopy showed a submucosal bulge at the dome with intact overlying mucosa. The bladder capacity was above normal limits. Transvesical biopsy was not done because the overlying mucosa was normal. The patient was told that the possibility of urachal carcinoma was high because of the solid mass with enhancement on contrast. The patient was also told about the possibility of percutaneous diagnosis with its associated low yield. The patient was also told that an excision biopsy of the mass can be curative, and because of high bladder capacity, bladder preservation surgery could be attempted. The patient wanted a single procedure for relief and hence opted for an excision biopsy with bladder preservation. Hence, the patient underwent partial cystectomy with en bloc resection of the urachus and umbilicus. Histopathology revealed cystic urachal remnants with high-grade musculo-invasive urothelial carcinoma (Figure 2). The margins on histology were clear and devoid of tumor. Postoperatively, the patient underwent three monthly cystoscopies and ultrasounds (USG) with an annual CT examination. The patient was doing well until the one-year follow-up, when CECT showed recurrence (Figure 3). The patient was again subjected to laparotomy with excision of the recurrent mass and bladder preservation, as the patient and her family were willing only for the bladder preservation protocol. Histology revealed that the margins were clear again. The patient received chemo-radiotherapy as per bladder protocol. At the two-year follow-up, the patient was doing well with no evidence of recurrence. The patient was subjected to cystoscopy every three months and USG imaging every three months, with advanced imaging done at one year. There was no evidence of local recurrence at all. But at two years, she developed a chronic, irritating cough. A check PET (positron emission tomography) scan at this time showed disseminated metastatic disease with no recurrence at the local site. The patient succumbed eventually due to the effects of metastatic disease.

## Discussion

The urachus, a vestigial structure, is located in the Retzius space between the transversalis fascia and the peritoneum. It has intramucosal, intramuscular, and supravesical segments. It has three distinct layers: luminal urothelium, submucosal connective tissue, and an outer smooth muscle layer. Neoplasms can occur in any layer.

The pathological varieties of urachal urothelial carcinoma are urothelial CIS (carcinoma in situ), non-invasive high-grade carcinoma, coexistent variety, invasive carcinoma (index case), and mixed adenocarcinoma (AC) with urothelial carcinoma [8]. 

Paner et al. suggested criteria for pathological confirmation of urachal carcinoma other than adenocarcinoma [9]. The system needs three definite criteria: location at the dome or anterior wall midline in the supravesical region up to the umbilicus, a tumor epicentre away from the bladder surface, and no primary tumor of similar morphology elsewhere except in the genitourinary tract. It also needs any 1 of the 3 supportive criteria mentioned below to label it as of urachal origin: a close relationship with urachal remnant structures, non-involvement of the bladder surface, and absence of cystitis cystica/cystitis glandularis.

Our tumor fulfilled all six criteria and hence was labelled as urachal urothelial carcinoma.

Next, we would discuss the staging systems used in this rare growth, as it is an important prognostic parameter. There are three staging systems used [6]. The most commonly used is the Sheldon system. Sheldon’s stage 1 is non-invasive urachal carcinoma, stage 2 is invasive but confined to the urachus, stage 3 is late-stage disease with extension to the abdominal wall, peritoneum, and viscera other than the bladder, and stage 4 is metastatic disease with spread to nodes and distant sites. Stages 1 and 2 are early-stage diseases. Ashley or Mayo’s system is a simplified modification of Sheldon’s, where stage 3 includes nodal disease, and stage 4 depicts metastatic disease. Ontario’s system has urachal confined disease classified as stage 1. Bladder involvement becomes stage 2. Contiguous fat involvement is stage 3. Peritoneal spread is stage 4. The index case fulfills the criteria of early-stage disease.

We would like to highlight some of the preoperative diagnostic dilemmas in this rare tumor. The urachal location makes it ideally suitable for ultrasound or CT. There was only one article in the literature that shed light on this aspect. Spataro et al. have emphatically stated that differentiation of infected thick-walled cysts from carcinoma may be difficult, and it is in these cases that fine needle biopsy with cytological examination of the fluid is helpful, but CT is highly diagnostic for a solid tumor as seen in our case [10]. Hence, we did not plan for a preoperative fine needle biopsy, as we felt that the risk of track seeding with a tumor may outweigh any benefit accrued.

Next, we would like to discuss the treatment. Historically, partial cystectomy with en bloc urachectomy cures 70% of patients. Further resection of the umbilicus is recommended, as it increases the cure rate to 81%, and the umbilicus has been shown to harbor tumors in 7% of cases [6]. The debate between radical and partial cystectomy has not been answered because of the rarity of cases. The present case highlights the fact that partial cystectomy is not therapeutic for early-stage malignancies if they are already musculo-invasive. We feel that in the presence of muscle invasion, radical cystectomy should be offered as the standard of therapeutic gold because of the aggressive nature of the tumor, and conservative bladder-preserving surgery should be abandoned in such cases. 

A literature search revealed a total of 13 reported cases of pure urachal TCC. Of these, six had no recurrences, and seven had recurrences. In the recurrent seven-group, five died. Hence, the overall survival is around 61.5% (8/13). Of these 13 cases, only four presented with early-stage disease. Histological grading was available in only one case [7]. The conclusion from the above studies was that higher presentation stage and positive margins are predictors of poor outcome. There is no mention of the impact of grading and microscopic muscle invasion on prognosis in early-stage disease like ours. The present case is the first reported case of high-grade muscle-invasive carcinoma presenting with early-stage disease. In our case, both grading and muscle invasion seem to have affected prognostication more than staging. In the present index case, though the surgical margins were negative, the patient had a recurrence. So, we feel that the surgical margin status does not affect the prognosis as much as the musculo-invasive status.

Hence, we propose that both grading and muscle-invasive disease have the same impact on prognosis in urachal TCC as that of bladder TCC. We feel that submucosal musculature involvement in urachal TCC is as equally important as that of detrusor involvement in bladder TCC. The occurrence of recurrent disease in our case infers that the presence of a musculo-invasive component warrants aggressive therapy in the form of radical surgery, irrespective of the stage of the tumor. Bladder-preserving protocols in such high-grade musculo-invasive tumors should not be recommended.

## Conclusions

The clinico-pathological features of urachal transitional cell carcinoma are not so distinct from bladder transitional cell carcinoma. Musculo-invasive transitional cell carcinoma of the urachus is a rare tumor. Contrast-enhanced CT is the diagnostic investigation of choice. Radical cystectomy with en bloc resection of the urachus and umbilicus is the treatment of choice if muscle invasiveness is present, irrespective of the stage and margin status. Urachal submucosal musculature involvement and high-grade are more important independent prognostic indicators than staging and positive surgical margins in this rare subset of tumors.
